# No. II. On the Medical Treatment of Insanity

**Published:** 1854-04-01

**Authors:** Forbes Winslow


### LETTSOMIAN LECTURES.

### No. II.

### ON THE MEDICAL TREATMENT OF INSANITY.

*Delivered before the Medical Society of London, April 7, 1852.*

By FORBES WINSLOW, M.D., D.C.L.

The purport of this lecture is the Medical Treatment of Insanity. It may be surmised that I have selected this subject with the view of submitting for your consideration, and through you to the profession, the particulars of a mode of treating the diversified morbid affections of the mind, original in its conception, *invariably* successful in its results, and based upon my own peculiar views as to the pathology of the disease. I am anxious, *in limine*, to disabuse your minds of these ideas. I lay no claim to any *exclusive* or *specific* mode of treating insanity. I possess no panacea, I have discovered no infallible medicine, no elixir, no drug that will

"Purge the mind of its thick-coming fancies,"

disperse the creations of the morbid imagination, restore the intellect to its just equilibrium, invigorate the judgment, give impetus and power to the paralysed volition, overpower the suicidal and homicidal impulse, elevate the depressed emotions, revivify the lost affections, or

"Chase away the furrow'd lines of anxious thought."

I should indeed be thankful if it were in my power to recommend to my professional brethren, any *specific* and *uniformly* efficacious course of medical treatment, likely to be followed by such happy results. Other motives and different feelings have influenced me in bringing this important and much-neglected matter before the profession, and have induced me to make it one of the "Lettsomian Lectures," which I have the privilege of delivering before this Society.

In considering the present aspects of the medical profession, I have been impressed by the conviction, that, as philosophers in search of truth, we have hitherto paid too little attention to the study of the science of therapeutics. Extraordinary talents, enlarged capacities, high attainments, profound knowledge, great power of continuous and laborious scientific investigation, indomitable and unflagging industry, united to habits of close and accurate reasoning, are devotedly and zealously engaged in the study of the different branches of our noble science. I ask, whether the great, the original, and truth-loving minds among us have investigated, in a manner proportionate to its vast importance, that section of our art which specially and exclusively relates to the *modus operandi* of medicines, and their therapeutic influence in the *actual cure of disease?* I feel reluctant to breathe a word, or to utter a syllable, which could in the slightest degree be supposed to convey the impression that I undervalued and underestimated those essential and interesting departments of the science of medicine, to the investigation of which so many highly-gifted men are devoting their talents and knowledge. The microscope has done much to enlarge the boundaries of science; it is an invaluable instrument in the hands of the scientific, experienced, and cautious philosopher, and the insight which it has afforded, and the light which it has reflected upon the minute anatomy of tissue, and into the nature of organic and pathological products and elements, have undoubtedly advanced considerably the science which we cultivate. The results so obtained have led to, and will ultimately be productive of, most important practical advantages. I say so much in this stage of my inquiry, to guard myself against the imputation of thinking lightly of these minute inquiries into the intimate nature of organic structure. I would not say a word to discourage the commendable zeal, industry, and patience of the microscopist, who toils

"From night to morn, from morn to dewy eve,"

in investigating the phenomena of matter, and who applies well-ascertained data to assist him in the elucidation of that mysterious and subtle principle which gives motion, animation, and intelligence to the grosser particles of our material organization. Admitting the great utility of the microscope, I would, placing my interrogatory in a suggestive form, ask, whether we have not, in these profound, intellectual, and necessary investigations, occasionally overlooked the great and ostensible vocation of the physician? The erudite anatomist—the learned physiologist—the accurate stethoscopist—the profound analytical chemist—the zealous microscopist, capable of accurately delineating the minute anatomy of tissue, or the physical character, weight, and quality of each essential organic element constituting its structure—will not, without the patient study of the phenomena of disease, and careful investigation of the *modus operandi* of the agents of the materia medica in certain morbid conditions of the system, make either a good or a successful physician. Have we not neglected the science of therapeutics? Have we devoted a sufficient degree of attention to the study of the specific action of medicine, under given conditions of bodily disease? Have we endeavoured to discover the most speedy mode of arresting the disorganizing process, assuaging suffering, prolonging the duration of life, and averting death, by the persevering administration of *physical* curative agents? It is

> "The wise physician, skilled our *wounds to heal,*"

who is represented by the bard of ancient days

> "As more than armies to the public weal."

Having in my previous lecture dwelt at considerable length upon the importance of watching the influence of the *morale* upon the *physique,* and having directed your attention to the invaluable mental remedies which the physician has at his command in the treatment of disease—in fact, to the subject of MORAL THERAPEUTICS,—I may be considered to be deviating from my original position, by bringing specially under your notice the subject of the *medical,* in juxtaposition to the *moral,* treatment of insanity. I hope, before I conclude, to satisfactorily establish that in urging this matter upon your serious attention, I am advocating no views in the remotest degree inconsistent or adverse to those propounded in my former lecture; or any that will militate against a legitimate use of moral means in the cure of the disordered affections of the mind.

It must be confessed that but little attention has been paid, by those possessing great opportunities for observation and practice, to the exhibition and action of physical remedial agents in the treatment of those abnormal conditions of nervous structure implicating the healthy action of the thinking principle. To what cause is this inexcusable apathy to be attributed? The neglect of the use of curative agents, on the part of those intrusted with the care of the insane, has not altogether arisen from an indisposition to make, by a persevering exhibition of appropriate medicine, an effort to re-establish the normal action of the brain and mind; but it is in the main the result—the necessary and inevitable consequence—of other causes, to which I shall refer. The doctrine promulgated by writers of celebrity—by men referred to and reverenced as our authorities and guides in this special department of medicine—that for the cure of insanity moral treatment is entitled to the highest rank, and to be deserving of the first consideration, has naturally tended to discountenance the administration of physical remedies in the treatment of insanity. We have been taught that *medical* ought to be subsidiary to *moral* means; and that any suggestion to remove a morbid mental impression by the aid of medicine, would indicate, on the part of the person making such a proposition, an inexcusable amount of ignorance, mental obtuseness, and obliquity! A recent writer on the subject of insanity exclaims, "When one man thinks himself a king, another a cobbler, and another that he can govern the world with his little finger, *can physic make him think otherwise?*"* Again: another author, in a work written to instruct the profession as to the treatment of the disorders of the mind, preposterously repudiates the idea of administering medicine for the cure of insanity, whilst the real nature of the mind remains unknown! He observes: "To prescribe for the mind, whilst *its nature remains a mystery*, is to prescribe for a *phantom!* As well might the mechanic attempt to regulate the multifarious operations dependent upon the agency of steam, by abstract discussion upon its nature, or to repair a fractured wheel, by directing his attention to the power that gave it motion, as for us to expect a successful result from remedies applied to an object the true nature and character of which we are wholly ignorant of; or of which, at least, we can only judge in its developments."† Alas! can we conceive more fatally paralyzing doctrines—opinions so antagonistic to all right views of the science of pathology, and so extremely detrimental to the advancement of therapeutics—so disheartening to those who feel anxious to bring the powerful agents of the

* Dr. E. Willis on Mental Derangement.

† "Practical Notes on Insanity," by F. B. Steward, M.D., p. 37.

materia medica to bear upon the treatment of this distressing form of disease.

Among the causes which have unfortunately given force and longevity to the idea that the administration of physical agents is of little or no avail in the treatment of the disorders of the mind, one holding the most prominent rank is the unphilosophical hypotheses which have been broached with the view of explaining the phenomena of insanity. To this source much of the fallacy, false induction, bad logic, and the neglect in reference to the use of remedial measures may be traced. Insanity has been considered to be a spiritual malady—a functional disease; to be an affection of the immaterial essence; to be a disorder of the soul, and not simply the result of a derangement of the material instrument of mind interfering with the healthy action of its manifestations. The brain has been supposed to be intact; not a fibre disturbed, not a vesicle altered, not a vessel overloaded: the encephalon has been imagined, in the severest forms of disturbed mind, to exist in all its integrity, so ridiculously absurd, so wildly unphilosophical, have been the notions entertained in reference to the proximate cause of insanity. This spiritual doctrine has naturally led to the conclusion—false in theory and destructive in practice—that for the alleviation and cure of the spiritual malady, spiritual remedies were the most important and essential. The clergyman instead of the physician was therefore summoned to the bed-side of the insane, and the bible and prayer-book displaced the physical remedies prescribed for the cure of the cerebral disorder.

In the earlier periods of the history of medicine, insanity was attributed to Divine wrath, demoniacal, Satanic, or malignant influence. It is a continuance in a belief of views somewhat analogous to these, but in a modified, less offensive, and different form, even in the present enlightened age, which has operated so disadvantageously in retarding the progress of cerebral pathology and therapeutic science. It may be said that a spiritual pathologist is a phenomenon—that the belief in the theory of insanity being an affection of the immaterial principle, is but an historical curiosity, a reminiscence of the dark ages. Alas! such is not the fact. I appeal to those whom I have the honour of addressing, whether a disposition does not exist among a considerable section of the profession to repudiate the idea of morbid action being invariably the result of some abnormal state of the organic tissue.

The common phrase, "functional disease," is but another designation for the spiritual hypothesis—it is but a phantom of the mind—a pathological enigma, having no actual existence apart from the active imagination which gave it birth. When we assert that the "functional" or "spiritual" theory will not bear the test of serious examination—that it is at variance with all *à priori* and *à posteriori* reasoning—that it stands in direct opposition to positive, well-recognised, undeniable data, we are met by the interrogatory, Can you demonstrate to us the specific character of the change induced in the nervous matter which it is alleged gives rise to mental derangement? and do not the scalpel and microscope of the morbid anatomist in vain endeavour to ascertain, in many cases of positive, violent, and unequivocal insanity, any appreciable structural lesion in the nervous matter, in its investing membranes, or organs in close association with the brain, sufficient to account satisfactorily for the morbid phenomena exhibited during life? One would really infer, from the reasoning and assertions of those who take these spiritual views, and who repudiate the idea of insanity ever being the result of a physical change in the condition of some portion of the brain or its appendages, that the encephalon has no specific functions allotted to it; that it is altogether a useless and supernumerary organ; that it was created for no wise purposes; and that, as far as the phenomena of mind were concerned, we could have done as well without as with the brain! If this organ be not the material instrument of mind—if it be not the media through which the spiritual portion of our nature manifests its powers—the centre of sensation—the source of volition—the seat of the passions—

"The dome of thought,—the palace of the soul"—

I ask, what *are* its functions, its specific uses and operations?—for what object was this most exquisitely organized and complicated structure formed?—why does it receive so large a proportion of the blood, and why is it so carefully protected from injury? These interrogatories naturally arise in the mind, when we hear so unphilosophical and so unphysiological a theory propounded with reference to the possibility of the mind being

*subject to disease apart from all derangement of the material organs with which it is so closely and indissolubly associated.* Can we conceive a more preposterous notion than that sanctioned by high authority, and which inculcates that the spiritual principle admits of being distorted, deluded, depressed, exaggerated, perverted, exalted, independently of any form of bodily disease, or modification of nervous matter?

Is it necessary that I should, in this advanced age of the science of physiology, stop to argue the question as to whether the brain be or be not the material organ of the mind? Unless we admit this fact, how many curious psychological and pathological phenomena must for ever remain mysteriously inexplicable? In infancy, when the brain is only partially developed, and but imperfectly organized, the mental faculties are obscurely manifested. As the infant approaches childhood, and the brain expands in volume, and the convolutions become more complex in character, the capacities of the mind become enlarged. In the middle period of life, when the brain is supposed to have attained its highest and perfect state of organization, we recognise the mind exercising its most elevated attributes. As we descend in the scale, we discover, in a ratio to the encroachment of age and the advancement of decrepitude, a proportionate diminution of mental vigour and astuteness; the faculty of attention and the powers of observation are less acute, the memory is incapable of retaining impressions, the judgment is often weakened, the temper capricious,—in fact, all the faculties of the understanding become (as a general rule) impaired. This mental decay slowly progresses until the

"Evening twilight of our existence,"

when we fall into the

"Sere and yellow leaf."

That rapid association of ideas and sense of the ludicrous which were wont to "set the table in a roar," are no longer manifested; the brilliant repartee, the gorgeous imagery, the poetic fancy which captivated, and the glowing and impassioned eloquence which enchanted, no longer exercise, like a magic spell, their influence over us. The mind at this period is incapable of any intellectual improvement: it feeds upon the past. The recollection of former scenes, however, in which it played a conspicuous part, continues vivid. In the evening, there is no memory of the occurrences of the morning; the brain appears to be incapable of receiving new impressions; ideas obtain no permanent hold of the mind, the intellect thus realizing the beautiful description recorded by Locke, who says, when speaking of the decay of the mind in old age,—"Ideas often die before us, and our minds represent to us those tombs to which we are approaching, where, though the brass and marble remain, yet the inscriptions are effaced by time, and the imagery moulders away."

Having considered this spiritual theory of insanity in an *à priori* point of view, what are the deductions which we are justified in making, looking at the *vexata questio* à posteriori? It has been frequently urged by those who discard the material hypothesis or explanation of the phenomena of deranged mind, that if insanity were the effect of brain disease, not only should we invariably find after death morbid changes in this organ, but we should detect some peculiar and specific alterations in the nervous matter, entirely distinct in their character from the ordinary lesions of structure detected in the more obvious diseases of the encephalon. With reference to the first position, I need only refer to the recorded opinions of all the great cerebral pathologists, from the great Morgagni down to modern writers, to establish beyond all question, cavil, or dispute, that in the great majority of cases of death after attacks of insanity, the brain, some of its important organic elements, or its investing membranes, are found in an abnormal morbid state. It is true that Esquirol somewhat encouraged the doctrine of the spiritualists, by asserting that in many instances of insanity no change in the nervous matter could be detected after the most careful scrutiny; but that high authority was known to have materially altered his views upon this point at a more advanced period of his life; and his later pathological investigations tended, I think, conclusively to establish that the nervous matter was always found modified in its structure after death from insanity. To this subject I have paid much attention, and have patiently endeavoured to ascertain what are the acknowledged opinions of those who have had opportunities of arriving at safe results, and whose names entitle everything which they have recorded to our profound deference and respect. I have carefully, scrupulously, and zealously analyzed no less than 10,000 cases of the various shades and degrees of insanity, related by Esquirol, Pinel, Foville, Georget, Guislain, Calmiel, Flourens, Bell, Haslam, Prichard, Solly, Burrows, Baillarger, Boismont, Abercrombie, Bennett, and other British, American, and continental authorities; and as the result of these pathological researches, I have no hesitation in declaring that I feel, as the natural effect of the influence of these well-ascertained data upon my own mind, amazed that there ever could have existed a shadow of a doubt as to the physical origin of insanity. The statistical facts to which I refer are not yet sufficiently matured and arranged to submit to the profession; but I may say that they satisfy my own mind, beyond all suspicion, of the *material cause of mental derangement.* I do not maintain that I am in a position to describe the peculiar and specific alterations which some allege to give origin to that derangement of the action of thought to which we apply the term insanity. Admitting such a discovery to be beyond the range of finite intelligence, it does not in the slightest degree militate against the material view just propounded. We find the functions of the eye, lungs, heart, stomach, liver, all deranged in a most marked manner, as the consequence, not of one peculiar specific affection of these organs, but of a variety of diseases essentially different in their pathological character, and only resembling each other in producing an altered action of the organic function of the part. Why should an important organ like the brain be exempt from the influence of the vital laws regulating the morbid action of other structures? and why should those who advocate the material origin of insanity be taunted and twitted because they are unable to discover an affection of the nervous matter *sui generis* in its character, and invariably discoverable in the brain in cases of death from mental aberration?

How often does death occur from apoplexy, convulsive disease, affections of the heart, stomach, from catalepsy, chorea, protracted hysteria, without evidencing any morbid condition of the structure, supposed to be implicated in the morbid process, appreciable to the eye of the pathologist; yet we are not sufficiently bold to maintain that catalepsy, apoplexy, epilepsy, disease of the heart, violent convulsions, severe gastric derangement sufficient to impede all nutrition, and persistent hysteria in all its Protean forms, can ever be viewed as strictly functional in their character, and capable of existing apart from any disease or abnormal state of the material tissue. But are we satisfied that in the cases of apparently functional disorder recorded by authorities of character and repute, the brain was accurately and scientifically examined—that the microscope aided the senses of the pathologist in his investigation? Was the brain, in all cases cited for the purpose of establishing that this organ was entirely free from all abnormal change, carefully macerated, weighed, and the different layers of the grey portion of the convoluted surface zealously scrutinized, in order to ascertain whether any change had taken place in its delicate structure? Was the chemical composition of the brain ascertained? Was the vesicular neurine minutely examined by means of a high microscopic power? Was it ascertained whether the blood was deprived of any of its essential and important constituents, and, as a consequence of such vitiated state, interfering with the healthy nervous nutrition? Were the blood-vessels of the brain removed and examined, with the view of ascertaining their calibre and condition of their coats? Was the state of the bones of the cranium, as well as the foramina, ascertained?

The spiritualists point with exultation to the cases recorded by Abercrombie and others, of extensive organic alterations having been found in the brain, which during life had not in the slightest degree, apparently, impaired or interfered with the normal action of mind; but if we carefully and scientifically investigate these instances, so often pompously and triumphantly paraded, I think we shall be compelled to admit they do not constitute data entitled to any weight in the solution of the important question at issue. It would be necessary for us to be informed upon good and unquestionable authority, of the precise character and locality of these alleged organic alterations—whether they were limited to the medullary, or extended to the cineritious portions of the cerebral matter; whether they were of slow or of sudden production; and also, whether the mind of the person having so great a degree of alleged disorganization discoverable in the brain after death, was carefully examined, and the actual condition of the mental powers satisfactorily ascertained. Positive, glaring, appreciable lunacy might, I readily admit, have been non-existent during life—the party need not *necessarily* have been insane, or guilty of any overt act of violence or extravagance sufficient to excite observation or compel restraint; but, nevertheless, the mind, in its *general operations*, might have been *considerably impaired and debilitated*, these affections having escaped notice, and not have been made matter of record. I am much disposed to consider that if the history of the cases narrated, of extensive disorganization of the brain without obviously implicating the faculties of the mind, were carefully and minutely examined, it would have been found in *every case that the intellect more or less suffered, although occasionally not to the extent of recognisable, positive, and clearly-defined insanity.* Without a knowledge of all these important particulars, the data referred to are, in a purely scientific point of view, entitled to but little consideration. I can imagine that considerable lesion of structure might exist, if confined to the medullary portion of the brain, without obviously or palpably deranging the intellectual operations; but *no morbid change can exist in the hemispherical ganglia without involving to some extent the operations of the mind.*

In considering this matter, we should not forget that the brain can accommodate itself to a considerable amount of actual loss of structure and organic disease, if the morbid changes be of slow and progressive growth. Again, it is necessary to ascertain whether, in these instances, both hemispheres of the brain were involved in the disease; for as the brain is a *dual* organ, it is possible for considerable structural disease to exist in one hemisphere, the opposite side remaining intact, without obviously interfering with the healthy action of the intellectual faculties. Again, it has been urged that insanity must, in many cases, be a functional and not an organic disease, because it has occasionally been cured by moral remedies alone; that a delusion has been dissipated by a joke, and an apparently fixed morbid idea has been dispersed by an ingeniously-contrived stratagem. Such illustrations of remarkable cures are undoubtedly upon record; but they no more establish that the disorder was spiritual and functional in its character, than the fact of a paroxysm of gout being overpowered by a sudden mental shock, an attack of convulsions arrested by calling into exercise the passion of fear, would justify us in concluding that the diseases referred to were functional and spiritual affections, having no relation to any morbid condition of the physical part supposed to be their seat.

Considering the subject practically, let us for a moment ask ourselves the question, what have been the consequences of the general belief in the spiritual and functional character of this disease? The lamentable effect has undoubtedly been, to discourage and discountenance the use of remedial measures; and the effect upon the public mind has, alas! been, to create the false impression that mental affections were not curable maladies, and that it was not in the power of the physician, by means of medicine, to administer to their relief. As the result of a too general belief in this sophistry—this dangerous fallacy—a vast amount of mental disease, particularly in its early and premonitory stage, is left to take its own uninterrupted course, until the unhappy sufferer has been placed beyond the reach of all curative agents. Why should the man who is conscious of the approach of mental infirmity—who feels his power of attention flagging, his volition becoming weakened, his affections perverted, and horrible fancies displacing healthy mental impressions—seek the aid of medicine, or fly to the physician for assistance, if he is taught to believe that the dark cloud which is gradually enshrouding his faculties is either the effect of a malignant spirit, the result of demoniacal influence, the consequence of the curse of the Almighty, or a disease entailed upon him as the punishment for his sins? "Madness," says Dr. Burrows, "is one of the *curses imposed by the wrath of the Almighty on his people for their sins, and deliverance from it is not the least of the miracles performed by our Saviour*"! I quote this passage to show what are the prevailing notions of the cause of insanity among the first authorities in this country.* Why should the relations and friends of those so unhappily afflicted seek the aid of medicine, when men of position and repute both publicly and privately propound such doctrines, and as a consequence discourage all physical treatment? Great and awful is the responsibility of those who thus thoughtlessly weaken the confidence of the public in the efficacy of physical curative agents in the treatment of insanity. "I was told," said a lady, "that medicine was of no avail in the affections of the mind. I went to the clergyman for assistance, but could obtain none. I have struggled for weeks heroically against the disposition to suicide, with the prayer-book in one hand, and the open razor in the other. Five times have I felt its keen edge at my throat, but a voice within me suddenly commanded me to drop the murderous instrument; and yet at other times the same voice urged me

* "Commentaries on Insanity," by Dr. Burrows.

desperately on to self-destruction. I knew I was ill—seriously ill—bodily ill; yet no one pointed out to me the right remedy for my horrible impulse, or recommended me to place myself in the hands of the physician." Such was the statement of a patient who voluntarily subjected herself to medical treatment, and was happily restored to health.

It is the prevalent opinion, even among persons otherwise well educated and intelligent, that the desire of self-destruction is in the majority of cases a mental act, unconnected with a disturbed condition of the bodily function, and incurable by any process of medical treatment; that the mental depression which is so generally associated with the suicidal tendency is an affection of the mind *per se*, the physical organization having no direct connexion with what is termed the *spiritual* impulse. This metaphysical view of the matter is fraught with much mischief, and, I have no doubt, has led to the sacrifice of many valuable lives. It is a matter of the highest moment that the public mind should be undeceived upon this point. Right views on this subject ought to be generally diffused. It is of consequence to establish the belief that the suicidal idea is *almost generally* connected with a morbid condition of the mind, and is often the only existing evidence of such an affection; that it is, with a few exceptions, universally associated with physical disorder, disturbing the healthy balance of the understanding; and that the bodily affection, which is, in nine cases out of ten, the *cause* of the mental irregularity, is easily curable by the judicious application of remedial means. The tendency of the spiritual or metaphysical view of the question is to create a distrust in remedial measures, and the poor man who is struggling against an almost overpowering desire to destroy himself is induced to neglect entirely his lamentable condition, under the belief that he is literally placed beyond the reach of curative agents, and that the only remedy for his mental suffering is death!

If a person in this unhappy state of mind is induced to believe that his mental despondency is but a *consequence* or *effect* of a disturbed bodily condition, influencing, either directly or indirectly, the natural and healthy operation of the brain and nervous system, and giving rise to perverted ideas—that his malady is curable, he may be induced to avail himself of the means which science has placed at the disposal of the physician, and thus be protected against his own insane impulses.

Where no disease is suspected, no remedy will be sought. Tell a man who has attempted to destroy himself that he is perfectly sane—that his judgment is sound—that his will is not perverted—that the impulse which urges him to the commission of suicide is not associated with any deviation from corporeal health—and you inculcate ideas not only fallacious, but most pernicious in their character and tendency. We might, with as much truth, tell a person playing with a lighted taper at the edge of a barrel of gunpowder, that his life is not in jeopardy, as to say to a person disposed to suicide that he is in the perfect enjoyment of health, and requires no moral or medical treatment. It may be laid down as an indisputable axiom, that in every case of this kind, bodily disease may, upon a careful examination, be detected. I never yet saw a case where a desire to commit suicide was present, in which there was not corporeal indisposition.

Having in the preceding portion of these observations endeavoured to establish what I conceive to be an important and necessary preliminary point, it is now my province to bring under notice a sketch, a mere *outline*, of my own views as to the pathology and medical treatment of insanity. Before referring to this part of my subject, I would premise that no right estimate can be entertained of the importance of these investigations unless we apply to the study of the diseases of the brain, and the cure of its disorders, the same enlarged and general principles which guide us in the investigation and treatment of the affections of other organic structures. An error of some magnitude has been committed by those who consider insanity to be a special, uniform, specific, and peculiar malady, justifying us in placing those so afflicted out of the ordinary nosological scale and sphere of medical practice. Again, it is necessary that we should, before being able to appreciate the effect of medical treatment, entertain just and enlightened views as to the CURABILITY of INSANITY. I now speak from a somewhat enlarged experience, from much anxious consideration of the matter, and I have no hesitation in affirming that, if brought within the sphere of medical treatment in the earlier stages, or even within a few months of the attack, insanity, unless the result of severe physical injury to the head, or connected with a peculiar conformation of chest and cranium, and an hereditary diathesis, *is as easily curable as any other form of bodily disease for the treatment of which we apply the resources of our art.* Can there be a more lamentable error, or a more dangerous, false, or unhappy doctrine than that urged by those who maintain that the disordered affections of the mind are not amenable to the recognised principles of medical science ?* I again declare it to be my positive and deliberately formed opinion, that there are few diseases of equal magnitude so susceptible of successful medical treatment in the incipient form as those implicating the normal action of thought. The existence of so vast an amount of incurable insanity within the wards of our national and private asylums, is a fact pregnant with important truths. In the history of these unhappy persons—these lost and ruined minds—we read, in many cases, recorded the sad, melancholy, and lamentable results of either a total neglect of all efficient curative treatment at a period when it might have arrested the onward advance of the cerebral mischief, and maintained reason upon her seat; or of the use of injudicious and unjustifiable measures of treatment under mistaken notions of the nature and pathology of the disease. In no class of affections is it so imperatively necessary to inculcate the importance of early and prompt treatment, as in the disorders of the brain affecting the manifestations of the mind. I do not maintain that our curative agents are of no avail when the disease has passed beyond what is designated the "curable stage." My experience irresistibly leads to the conclusion that we have often in our power the means of curing insanity, even after it has been of some years' duration, *if we obtain a thorough appreciation of the physical and mental aspects of the case, and perseveringly*

* "You do not pretend to *cure* insanity!" exclaimed a gentleman of considerable intelligence to me, whilst detailing the particulars of a distressing attack occurring in a member of his own family; "for," he continued, "I heard Dr. —— positively declare, in a public lecture, that '*he lamented to be obliged to say, that in the cure of insanity, little or no good resulted from medical treatment.*'" Sad and fatal doctrine! Whilst recently visiting Bethlehem Hospital, to see, at the request of their friends, two patients in that establishment, I heard a foreigner who had been inspecting the asylum observe to Dr. Wood (the then resident medical officer of the establishment), whilst talking of the medical treatment of insanity, that it was quite a mistake to have a portion of the asylum set apart for the "incurable patients." "*The word 'incurable,'* in reference to insanity," he continued, "*should never be used.*" I would much prefer pinning my faith to the doctrine of the foreign than to that of the English physician, who attempted to weaken our confidence in the curability of insanity by means of medicine.

*and continuously apply remedial measures for its removal.* I cannot, however, dwell too strongly upon the vital necessity of the early and prompt exhibition of curative means in the incipient stage of mental derangement:—

> " Principiis obsta : sero medicina paratur
> Cum mala per longas convaluêre moras."—OVID.

It becomes necessary, before proceeding to the consideration of the practical division of my subject, that I should briefly refer to the morbid anatomy of the brain in insanity. It is not my intention to cite the conflicting opinions of writers of repute in reference to this section of pathology; neither shall I attempt to reconcile the varied and contradictory statements of eminent pathologists who have investigated this important subject.

With these prefatory observations, I will concisely submit to you the conclusions to which I have arrived in relation to this much-vexed question. I believe insanity (I am now referring to persistent insanity, not those transient and evanescent forms of disturbed mind occasionally witnessed) to be the result of a *specific morbid action of the hemispherical ganglia, ranging from irritation, passive and active congestion, up to positive and unmistakeable inflammatory action.* This state of the brain may be confined to one or two of the six layers composing the hemispherical ganglia; but all the layers are generally more or less implicated, in conjunction with the tubular fibres passing from the hemispheres through the vesicular neurine. This specific inflammation, from its incipient to the more advanced stage, is often associated with great vital and nervous depression. It is, like analogous inflammations of other structures, not often accompanied by much constitutional or febrile disturbance, unless it loses its specific features, and approximates in its character to the inflammation of active cerebritis or meningitis. This state of the hemispherical ganglia is frequently conjoined with active sanguineous circulation or congestion, both of the substance of the brain and its investing membranes. The morbid cerebral pathological phenomena—viz. the opacity of the arachnoid, the thickening of the dura mater, its adhesions to the cranium, the depositions so often observed upon the convoluted surface of the hemispheres, and on the meninges, the hypertrophy, scirrhus, the cancerous affections, the induration, the depositions of bony matter in the cerebral vessels and on the dura mater, the serous fluids in and the ulcerations upon the surface of the ventricles, the alterations in the size, consistence, colour, and chemical composition of the vesicular neurine and fibrous portion of the brain —are all, in my opinion, the *results*, the *sequelæ*, more or less, of that specific inflammatory condition of the hemispherical ganglia to which I have referred. It does not necessarily follow that the *fons et origo mali* of insanity is invariably to be traced to the brain. The preliminary morbid action is often situated in the heart, stomach, liver, bowels, uterus, lungs, or the kidneys, the brain being only *secondarily* affected; nevertheless, in all cases inducing actual insanity, the hemispherical ganglia are involved in the morbid action. The most recent pathological doctrine propounded to explain the phenomena of insanity—I refer to the views of a recent writer*—that derangement of mind is the effect of "*loss of nervous tone,*" and that this loss of nervous tone is "*caused by a premature and abnormal exhaustibility of the vital powers of the sensorium*"—conveys to my mind no clear, definite, or precise pathological idea. It is true that we often have, in these affections of the brain and disorders of the mind, "loss of nervous tone," and "exhaustion of vital power;" but, to my conception, these are but the *effects* of *a prior morbid condition of the encephalon*, the *sequelæ* of specific inflammation of the hemispherical ganglia. To argue that insanity is invariably and exclusively the result of "loss of nervous tone," is to confound cause and effect, the *post hoc* with the *propter hoc;* and would, as regards therapeutical measures, act as an *ignis fatuus*, and allure us as pathologists from the right and legitimate path. I feel anxious that my views upon this important subject should be clearly enunciated, and not open to misconception. I think much mischief has arisen from a belief in the existence of *active ordinary cerebral inflammation* in cases of insanity, for it has led to the adoption of treatment most destructive to life, and has seriously interfered with the permanent restoration of the reasoning powers. Nevertheless, insanity is *occasionally* complicated with acute cerebral symptoms sufficient to justify us in the cautious use of somewhat active measures for its removal. We must avoid the fatal error of a too rapid process of generalization, and be careful of not looking to symptoms instead of to the disease itself, and of permitting ingenious and well-constructed

* Dr. H. Munro.

*à priori* theories of the nature of insanity to dazzle our imaginations, and abstract the mind from the steady and patient investigation of pathological science, and individual cases of disease. If we allow our judgment to be warped by the inflammatory theory on the one side, (I am now speaking of *ordinary*, not of *specific* inflammation,) and conclude that the excitement of mania is to be subdued by copious depletion or the administration of antiphlogistic measures,—or if, on the other hand, we adopt the speculative opinions of those who believe that in every case of insanity, irrespectively of its origin, its progress, or its character, there exists "mere loss of nervous tone," caused by a "premature abnormal exhaustibility of the vital powers of the sensorium,"—how lamentably shall we be misled as to the real character of the disease, and in the application of our therapeutic agents? These circumscribed and partial views of the pathology of insanity, often, alas! lead to serious solecisms in practice. In ninety per cent. of the cases of acute mania, there is found in the brain and its meninges a state of sanguineous congestion, particularly of the hemispherical ganglia, combined with alterations in the grey nervous matter. In forming an opinion of the actual pathological condition of the cerebral substance, we should remember that, particularly in public asylums, it is a rare occurrence for recent cases to be admitted; that the acute and sub-acute active cerebral conditions have subsided, and the disease has assumed a chronic form, before the patient is examined and placed under treatment; consequently many deductions recorded by pathologists have been based upon the study of chronic, and not of acute, mania. A large per-centage of the cases, before admission into our national asylums, have passed through the primary and acute stages, and have probably been subjected to medical treatment. This fact must never be lost sight of in forming our opinion, not only of the nature of the disease itself, but of the medical treatment necessary for its cure. In private practice the acute forms of insanity are often met with; but even with the advantages which the physician in general practice can command, of investigating the earlier stages of deranged mind, he often discovers that the mental affection has been allowed to exist and slowly progress for a considerable period, no treatment, either medical or moral, having been adopted for its removal. In the incipient forms of insanity, particularly when it manifests itself in plethoric constitutions, has been sudden in its development, is the result of physical causes, and is connected with the retrocession of gout, or is rheumatic in its character, there can be no doubt the nature of the change induced in the brain is more allied to that of inflammation than that of nervous exhaustion. The attacks from the slow and insidious operation of moral causes are less likely to be accompanied by active cerebral symptoms. In many instances the maniacal excitement is *asthenic* or *atonic* in its character, resembling the delirium of the last stages of typhus fever.

The most simple classification of insanity, the one I think best adapted for useful and practical purposes, is its division into the *acute* and *chronic* forms; the insanity ushered in by *excitement* or by *depression*, into *mania* and *melancholia—amentia* and *dementia.* The minute divisions and subdivisions, the complicated and confused classification taught by lecturers and found detailed in books, may serve the ostentatious purpose of those desirous of making a pompous display of scholastic and scientific lore, but I think they have tended to bewilder and obscure the understanding, and lead the student in search of practical truth from the investigation of the *disease itself* to the study of its *symptoms*, and to the consideration of unessential points and shades of difference. Adhering to this division of the subject, each form should be viewed in relation to its *complications*, as well as to its *associated diseases.* Among the former are epilepsy, suicide, homicide, paraplegia, hemiplegia, and general paralysis. The associated diseases implicate the lungs, heart, liver, stomach, bowels, kidney, bladder, uterus, and skin.

Before adverting to the preliminary examination of the patient supposed to be insane, and suggesting rules for arriving at an accurate prognosis in these cases, I would premise that those inexperienced in the investigation of this class of cases would often arrive at false and inaccurate conclusions, if they were not cognizant of the fact, that the insane often describe sensations which they have never in reality experienced, and call attention to important symptoms which have no existence except in their own morbid imaginations. A patient will assert that he has a racking headache, or great pain and tenderness in the epigastric region, both symptoms being the fanciful creations of his diseased mind. This is particularly the case in the hysterical forms of insanity, in which there always exists a disposition to pervert the truth, and exaggerate the symptoms. Again, serious bodily disease may be present, the patient not being sufficiently conscious to comprehend the nature of the questions asked, or able to give intelligible replies to the anxious interrogatories of the physician. Insanity often masks, effectually obscures, other organic affections, the greater malady overpowering the lesser disease. When Lear, Kent, and the Fool, are standing alone upon the wild heath, exposed to the merciless pelting of the pitiless tempest, Kent feelingly implores the king to seek shelter from the "tyranny of the open night," in an adjoining hovel. It is then that Lear gives expression to the great psychological truth just enunciated—

> "Thou think'st 'tis much, that this contentious storm
> Invades us to the skin: so 'tis to thee;
> But *where the greater malady is fixed,*
> *The lesser is scarce felt;*
> * * * * The tempest *in my mind*
> Doth from my senses take *all feeling else*
> *Save what beats there.*"

Disease of the brain may destroy all apparent consciousness of pain, and keep in abeyance the outward and appreciable manifestations of other important indications of organic mischief. Extensive disease of the stomach, lungs, kidneys, bowels, uterus, and heart, has been known, during an attack of insanity, to progress to a fearful extent, without any obvious or recognisable indication of its existence. Insanity appears also occasionally to modify the physiognomy and symptomatology of ordinary diseases, and to give them peculiar and special characteristic features.

Again, it is necessary for the physician to watch the operation of medicine in masking important diseases. The different forms of narcotics, if given in heroic doses, often mislead us in our estimate of the nature of bodily diseases not directly connected with the mental affection. In the examination of these cases the most essential preliminary matters of inquiry have relation to the age, temperament, previous occupation, and condition in life of the patient. It will be necessary to ascertain the character and duration of the attack; whether it has resulted from moral or physical causes; is of sudden, insidious, or of slow growth; whether it has an hereditary origin, is the effect of a mental shock, or of mechanical injury; whether it is the first attack, and, if not, in what features it differs from previous paroxysms. It will also be our duty to ascertain whether the insanity be complicated with epilepsy, paraplegia, or hemiplegia, or with suicidal and homicidal impulses. If any prior treatment has been adopted, we must inform ourselves of its nature; and also ascertain whether the patient has suffered from gout, heart disease, rheumatism, cutaneous affections, or syphilis? It is important to obtain accurate information in relation to the condition of the uterine functions, and to ascertain the state of the moral affections. We should also inquire whether the patient has been suspected of habits of self-abuse. Having obtained accurate information upon these essential points, our own personal observation will aid us in ascertaining the character of the mental disturbance; the configuration of the head, chest, and abdomen; the gait of the patient, the degree of sensibility and volitional power manifested; the state of the retina, the action of the pulse, the composition of urine, and temperature of the scalp and body generally; the condition of the skin and chylo-poietic viscera; the action of the heart, lungs, and nature of any existing disease of the uterus. If a patient complains of any local mischief, however imaginary it may appear to be at the time, it is essentially necessary that we should clearly satisfy our minds upon the point, before dismissing it as not entitled to serious investigation. A patient once bitterly complained of retention of urine; upon examination, the bladder, was found to be distended, and the man had passed no urine for twenty-four hours. I was about to introduce a catheter, when the patient burst into a fit of laughter, and immediately emptied his bladder. Esquirol relates a case of a merchant, who, whilst suffering from melancholia, declared that some foreign body was sticking in his throat. No notice was taken of this supposed fanciful idea. The patient died, and an ulcer was discovered at the upper third of the œsophagus. A patient complained of devils being in his stomach and bowels, and declared that they were acted upon by electric, magnetic agencies. After death he was found to have scirrhus of the stomach, and chronic inflammation of the bowels. A patient refused to eat; he said he could not swallow his food without great pain. As he had exhibited other symptoms of a disposition to suicide, it was thought by myself and others, that his obstinate refusal of food was associated with ideas of self-destruction. He died, and at the post-mortem examination a stricture in the pylorus was discovered.

These illustrations, and they could easily be extended, will prove the importance of paying minute attention to particular delusions, with the view of ascertaining whether they have not an *actual* physical origin.

The *prognosis* in cases of insanity will mainly depend upon the duration of the attack, its character and origin, and the diathesis of the patient. The prognosis is generally unfavourable if the disease be hereditary—if the symptoms are similar in character to those exhibited by other members of the family when insane. Insanity, accompanied by acute excitement, is, *cæteris paribus*, more easy of cure than when it has been of slow and gradual growth, and is marked by great mental depression. The prognosis is favourable in cases of puerperal mania; it is unfavourable when there exists a want of symmetry between the two sides of the head, with small anterior and large posterior cerebral development. Any great inequality in the cranial conformation would be a suspicious indication. The existence of any malformation in the development of the chest is also an unfavourable sign, and would induce us to give a guarded prognosis. Dr. Darwin says, when a person becomes insane, who has a small family of children to absorb his attention, his prospect of recovery is but small, as it establishes that the maniacal hallucination is more powerful than those ideas which ought to interest the patient most. The prognosis is unfavourable when patients are under the morbid delusion that they are poisoned, and constantly complain of suffering internally from peculiar sensations. Religious delusions are more difficult to eradicate than other morbid impressions. The age of the patient will materially guide us in forming a correct prognosis. Hippocrates says the insane are not curable after the fortieth year; Esquirol maintains the greater portion recover between the ages of twenty and thirty; Haslam between the ages of ten and twenty. As a principle, we may conclude that the probability of recovery in any given case is in proportion to the early age, physical condition, and duration of the attack. When a patient has youth and a good constitution to aid him, and is advantageously placed, having at command remedial measures, and is excluded from all irritating circumstances, the prognosis may be considered favourable. I have seen patients after the advanced age of sixty and seventy recover; and cases of cure are upon record, where insanity has existed for ten, fifteen, and twenty years. In forming our prognosis, it is important to ascertain the educational training of the patient. Has he been in the habit of exercising great self-control? Has his mind been well disciplined? Has he kept in abeyance the passions, or have the emotions and impulses of his nature obtained the mastery over him? He who has been taught to practise self-denial and self-control in early life is, *cæteris paribus*, in a more favourable position for recovery than he who has permitted himself to be the willing and obedient slave of every wild passion and caprice. Insanity, accompanied with criminal propensities, is said to be incurable, because, as Ideler urges, such patients "cannot bear the torments of their consciences, and relapse into the stupefaction of insanity to flee from the consciousness of their guilt."* The prognosis is unfavourable when the insanity is complicated with organic disease of the heart and lungs, with deafness, and paralysis in any of its forms.† Lesions of the motor power are very unfavourable indications. Great impairment of mind, accompanied with delusions of an exalted character, and associated with paralysis, is generally incurable. Esquirol says, epilepsy, if associated with insanity, places the patient beyond all prospect of cure. I should be loth to adopt this sweeping condemnation. I have seen attacks of epilepsy, combined with mental derangement, recover; although, I admit, they constitute a difficult class of cases to manage. Epileptic vertigo, the *Petit-Mal* of the French, is generally more disastrous in its effects upon the powers of the mind than other forms of epilepsy. The prognosis in these cases is generally unfavourable.

In submitting for your consideration a few *general principles of medical treatment*, I would premise, that, in a lecture like the present, it would be impossible to develop, in anything like detail,

* "No disease of the imagination is so difficult of cure as that which is complicated with guilt; fancy and conscience then are interchangeably upon us, so often shift their places, that the illusions of the one are not distinguished from the dictates of the other. If fancy presents images not moral or religious, the mind drives them away when they give it pain; but when melancholic notions take the form of duty, they lay hold of the faculties without opposition, because we are afraid to exclude or banish them: for this reason the superstitious are always melancholy, and the melancholy always superstitious."—Dr. Johnson. *Rasselas.*

† "Deafness is not of itself a symptom of insanity, but it is often a concomitant, and their combination forms incurable insanity. The reason probably is, that the same cause which destroys the hearing, or affects the auditory nerve, extends also to the brain itself."—Dr. Brigham.

the special and particular class of remedial agents adapted for all forms of deranged mind. My time will only admit of generalizing this subject, and of directing attention to some of the more prominent phases of insanity, and those which present to us the greatest obstacles and difficulties in their management.

In regard to the treatment of acute mania, the important and much litigated question among practitioners of all countries, is that relating to the propriety of depletion. Need I refer to the conflicting and contradictory opinions entertained by eminent writers on this important and much-vexed therapeutical point? Whilst some practitioners of great repute and enlarged experience fearlessly recommend copious general depletion for the treatment of insanity, and cite cases in which this practice has been attended with the happiest results, others, equally eminent, whose opinions are as much entitled to our respect, fearlessly denounce the lancet as a most fatally dangerous weapon, and shudder at the suggestion of abstracting, even locally, the smallest quantity of blood! In avoiding Scylla, we must be cautious of being impelled into Charybdis. The error consists in a vain effort to discover a *uniform mode of treatment, and attempting to propound some specific mode of procedure adapted to all cases.* He who maintains that bloodletting is never to be adopted in the treatment of mania, without reference to its character, its origin, the peculiar constitution of the patient, and the existence of local physical morbid conditions, which may be materially modifying the disease, and giving active development to morbid impressions, is not a safe practitioner. Neither would I confide in the judgment and practice of the physician who would, in every case of violent maniacal excitement, attempt to tranquillize the patient and subdue excitement by either general or local depletion.

In attacks of insanity, when the symptoms are acute, the patients young and plethoric, the habitual secretions suppressed, the head hot and painful, the eyes intolerant of light, the conjunctivæ injected, the pupils contracted, the pulse rapid and hard, and the paroxysm sudden in its development, *one* general bleeding will often arrest the progress of the cerebral mischief, greatly facilitate the operation of other remedies, and ultimately promote recovery. In proportion as the symptoms of ordinary insanity approach those of phrenitis, or meningitis, shall we be justified in the use of general depletion. Although it is only occasionally, in instances presenting peculiar characteristic features—cases occurring in the higher ranks of life, where the patient has been in the habit of living *above par*, and is of a sanguineous temperament—that we are justified in having recourse to the lancet, there is a large class of recent cases presenting themselves in the asylums for the insane, both public and private, in the treatment of which we should be guilty of culpable and cruel negligence, if we were to omit to relieve the cerebral symptoms by means of the *local* abstraction of blood. It is, alas! the fashion and caprice of the day to recklessly decry the application of cupping-glasses or of leeches in the treatment of insanity, in consequence, I think, of the slavish deference shown to the opinions of a few eminent French pathologists, who have, by their indiscriminate denunciation of *all depletion*, frightened us into submission, and compelled us to do violence to our own judgment. The local abstraction of blood is, in the hands of the discreet and judicious practitioner, *a powerful curative agent;* and yet it is the practice of some men, and men, too, of position, to discard altogether the remedy!

I will briefly refer to the kind of case in which the local abstraction of blood will be found most beneficial, if proper regard be had to the temperament, constitutional condition, and the local circumstances modifying the character of the attack. In insanity, when the exacerbations occur at the menstrual period, leeches to the vulva and thighs, with the use of the foot-bath and the exhibition of aloetic purgatives, will be attended by the most favourable results. In irregular and obstructed menstruation, the local abstraction of blood will be very serviceable. In suppressed hæmorrhoids, leeches to the neighbourhood of the sphincter ani will act beneficially by unloading the hæmorrhoidal vessels, and thus relieve the brain of undue excitement. In cases of nymphomania, leeches to the vulva are indicated, and have been known to produce great benefit. In cases of intermittent insanity, the paroxysm may often be cut short by relieving the overloaded state of the vessels of the head by means of cupping or the application of leeches. In some instances, I have applied leeches to the Schneiderian membrane, particularly for the treatment of insanity occurring in early life, and connected with conduct evidently the effect of cerebral irritation. I have seen this mode of procedure of essential benefit in persons of plethoric constitution and of sanguineous temperament. Occasionally the insanity is found to be associated with active visceral disease, or with hypertrophy and other affections of the heart. Under these circumstances, when there exists great tenderness over the region of any of the visceral organs, and we are satisfied, by a careful stethoscopic examination, that hypertrophy of the heart is present, leeches applied over the seat of the local mischief, conjoined with other appropriate treatment, will materially aid us in subduing the maniacal affection. In cases of illusions of hearing, or of vision, it will often be necessary to apply leeches behind the ears, or over the superciliary ridges. I have known this practice entirely remove the morbid illusions which had been embittering the patient's life.

But apart entirely from the local affections to which I have referred, for the treatment of idiopathic insanity, apparently without any complications, or modified by any of the associated diseases, the careful and temperate local abstraction of blood, when general depletion is inadmissible, will often materially shorten the duration of an attack and restore the mind to a healthy condition. I am anxious to record my favourable opinion of this mode of treatment, because I have witnessed so many sad results from an opposite timid and reprehensible neglect of the means placed within our power for the treatment of the varied forms and degrees of mental derangement. Sad consequences have undoubtedly followed the indiscriminate use of depletory measures. The presence of violent mental excitement has occasionally led the practitioner to the conclusion that the disease was of an active character; and in the attempt to allay the undue cerebral excitement by means of antiphlogistic measures, the patient has sunk into incurable and hopeless dementia! But whilst recognising an *anœmic* class of case, where great excitement is often associated with loss of nervous and vital power, we must be cautious in permitting serious disease to be creeping stealthily on in the delicate structure of the brain, no effort being made to relieve the congested cerebral vessels or inflamed nervous tissue, until serious disorganization has taken place in the vesicular matter, and the patient is for ever lost. In the treatment of acute mania, the remedy next in importance to cautious depletion is that of *prolonged hot baths.* To Dr. Brierre de Boismont, of Paris, at whose excellent institution I first witnessed the application of this remedial agent, the profession is indebted for reviving a practice which had long fallen into disrepute. In the treatment of acute mania, the prolonged hot baths will be found of the most essential service. Dr. Brierre de Boismont has recorded the history of sixty-one out of seventy-two cases that were subjected to this mode of treatment. Three-fourths of this number were cured in a week, and the remainder in a fortnight. The patients remain from eight to ten and fifteen hours in warm baths, whilst a current of cold water is continually poured over the head; the temperature of these baths is from 82° to 86° Fahr.; the affusions 60° Fahr. Among the therapeutic effects of these baths, Dr. B. de Boismont reckons a diminution of the circulation and respiration, relaxation of the skin, alleviation of thirst, the introduction of a considerable quantity of water into the economy, an abundant discharge of limpid urine, a tendency to sleep, a state of repose. This mode of treatment is said to be inadmissible in cases of periodic intermittent mania, in insanity beginning with great mental impairment, or associated with epilepsy or general paralysis. The result of my own experience of this plan of treatment has produced a very favourable impression upon my mind, and I think it is entitled to a fair trial in all our asylums where recent cases are admitted.

In some forms of acute mania it is desirable, as a substitute for depletion, to diminish the activity of the circulation by the exhibition of nauseating doses of the tartrate of antimony; it may be serviceably combined with the tinctures of digitalis and hyoscyamus. This remedy, however, requires close watching, as it often has been known to suddenly reduce the vital powers to a low ebb, and extinguish life. It will be found beneficial in proportion to the recent character of the case and the positive activity of the cerebral circulation. The tincture of digitalis was formerly in great repute as an anti-maniacal remedy; the experience of late years has not encouraged us in administering it in the doses prescribed by some of the old writers; nevertheless, it is a useful agent, and occasionally proves a valuable auxiliary in the hand of the practitioner who carefully observes its therapeutic operation.

For the cure of the acute forms of insanity, the douche bath has been much lauded; but this remedy is now rarely used in

British asylums. I have occasionally seen benefit derived from its exhibition, but great caution is required in its use. A patient has been subjected, whilst in a paroxysm of acute delirium, to the douche bath, and has sunk almost immediately into incurable idiocy! The physical shock has occasionally been known to produce a good moral impression. For illustration: a patient imagined himself emperor of the world, and would not allow any one to address him by any other title. The immediate application of the douche bath destroyed his idea of royal dignity, and he was willing to admit that he had never been, nor was at any time, a regal personage. A few hours subsequently the delusive impression returned in all its original force; the douche bath was again had recourse to, and a second time the morbid impression vanished; by a series of baths he was restored to sanity, and after his complete recovery, when the particulars of his case were placed before him, he observed, 'Why did you not whip me, and beat this nonsense out of my head? I wonder how you could have borne with my folly, or I have been guilty of such contemptible arrogance and obstinacy.' As a substitute for the douche, the shower bath is often used with great benefit, particularly in certain forms of melancholia, associated with nervous depression and general debility. In cases of melancholia, or other kinds of chronic insanity connected with a congested state of the liver, the nitro-muriatic bath will occasionally do much good. In a few instances I have noticed marked benefit from the use of Bertolini's sedative bath, composed of henbane two pounds, and equal parts of hemlock and cherry laurel leaves, well infused in a sufficient quantity of hot water. But the simple hot bath, in certain conditions of the nervous system, particularly in some forms of suicidal mania, is of the utmost benefit. A warm bath a short period before retiring to rest, bathing the head at the same time with cold water, particularly if the scalp be unnaturally hot, will often ensure a quiet and composed night, when no description of sedative, however potent its character and dose, would influence the system.

In the early stages of insanity, and throughout its whole course, the bowels are often in an obstinately constipated condition. The concentration of nervous energy in the brain interferes with that supply which should proceed to other structures; consequently there appears to be a want of healthy sensibility in the mucous membrane of the bowels, and an interruption to the peristaltic action of the intestinal canal. There is no class of agents which acts so certainly and effectually, in relieving the mind when under the influence of depressing emotion, as cathartics. The ancients considered hellebore as a specific in certain forms of melancholia. In the hands of modern practitioners, this drug has not been found to merit the high encomiums which have been passed upon it. It is important in every case of insanity, but particularly in the acute stages of mental derangement, to act powerfully upon the bowels by means of a succession of brisk purgatives. The bowels are often found gorged with fœcal matter, and immediate relief often follows the administration of two or three doses of calomel and colocynth, or of croton-oil. It will often be necessary to assist the operation of the cathartics by means of enemata. In hysterical and some other forms of insanity there is frequently a disposition on the part of the patient resolutely to resist the calls of nature, and, knowing this peculiarity, we must carefully watch the condition of the bowels, otherwise serious mechanical obstructions may ensue, followed by intractable diseases of the rectum.

Insanity is often associated with gastric and intestinal disease, with an irritable condition of the mucous membrane of the alimentary canal; and, in such cases, although it is important to relieve the bowels and prevent them from being constipated, we must bear in mind that the injudicious exhibition of irritating drastic cathartics may aggravate the mental disease, by increasing the gastric and intestinal irritation, and thus do permanent and irremediable mischief. Much injury may arise from the indiscriminate administration of cathartics. In insanity associated with menstrual obstructions, it will be necessary to exhibit the class of purgatives known to act specifically upon the lower bowel; consequently aloetic cathartics, such as the compound decoction of aloes, and the compound galbanum pill, are found of most service. In plethoric conditions of the system, when there is a marked determination of blood to the head, no medicine will relieve so speedily as active doses of the compound powder of jalap.

In the treatment of insanity, the class of medicines termed *sedative* play an important part. If exhibited with judgment, the most gratifying results often follow *their continuous and*

*persevering administration.* The sedative treatment of insanity is a subject of itself, and I quite despair of touching even upon the confines of the many interesting and important points involved in the consideration of this division of my lecture. In insanity unassociated with active cerebral circulation, congestion, or paralysis, or after the head symptoms have been relieved by the local abstraction of blood and the administration of appropriate medicine, the exhibition of sedatives will be followed by the most beneficial results. In recent cases they are generally inadmissible, except in delirium tremens and puerperal insanity, and other forms of derangement analogous in their pathological character and symptoms to these affections. In chronic insanity, in melancholia unconnected with abdominal repletion, or visceral disease, the persevering use of sedatives in various combinations will often re-establish sanity, when no other course of treatment would be successful in dispelling the illusive impressions, or raising the drooping and desponding spirits. Battley's solution, the tincture of opium, the meconite, acetate, and hydrochlorate of morphia, the preparations of hyoscyamus, conium, stramonium, camphor, hops, aconite, ether, chloroform, hydrocyanic acid, hydrochloric ether, Indian hemp, are all of great and essential service, if administered with judgment and sagacity. In suicidal insanity, when local cerebral congestion is absent, and the general health and secretions are in good condition, the meconite and hydrochlorate of morphia often act like a charm, if *uninterruptedly and perseveringly given* until the nervous system is completely under their influence. I have witnessed the most distressing attacks of suicidal mania yield to this treatment, when every other mode of procedure had failed. I could cite the particulars of numerous cases of this form of insanity radically cured by the occasional local abstraction of blood from the head, the administration of alteratives, the warm bath, and sedatives. In the exhibition of this powerful curative agent, our success will often depend upon a *ready adaptation of the form of sedative to the description of case in which it may be deemed admissible, and a judicious admixture of various kinds of sedatives.* I do not think we pay sufficient attention to this fact. I have often seen an apparently incurable and unmanageable case yield to a combination of sedatives, which had resisted the operation of any one or two when given separately. The extract of conium is often of service in cases of insanity combined with epilepsy; conjoined with mineral tonics, conium is occasionally of benefit, particularly in melancholia connected with chronic disease of the digestive organs and with neuralgia. In cases of uterine irritation, I have seen great good result from the combination of hops, camphor, and hyoscyamus. In illusions of vision, belladonna, commencing with quarter-grain doses, will be found a useful remedy. In insanity complicated with dysmenorrhœa, the combination of camphor with hyoscyamus, opium, or conium, may be given with great advantage. The hydrochlorate of morphia, in union with dilute hydrochloric acid, is said to be useful in cases where the sedative treatment is desirable. I am often in the habit of exhibiting sedatives and tonics in combination, particularly conium with iron, opium with quinine, or with the infusion or compound decoction of cinchona. In debility, with irritability of the nervous system, accompanied by restlessness, Battley's solution, with the preparations of cinchona, will often prove of great benefit. The tincture of sumbul I have occasionally administered, and I think with advantage, in paroxysmal or convulsive forms of insanity. I have given it to the extent of one or two drachms for a dose. In hysterical derangement, the tincture of Indian hemp will occasionally allay the excitement, and produce sleep more rapidly than any other form of sedative. The valerianate of zinc has not answered the expectations of those who have spoken so highly of its medicinal virtues. Tincture of opium with camphor, and the tartrate of antimony, is an excellent combination in cases of doubtful cerebral congestion. Tincture of hops, in doses of from one to four drachms, may be necessary, when no other formulæ are admissible. As mild forms of sedative, compound ipecacuanha powder, extract of lettuce, and the syrup of poppies, are occasionally recommended; a good substitute for Dover's powder is a pill composed of opium, ipecacuanha, and soap.

The more chronic forms of insanity, particularly melancholia, are occasionally difficult of cure. Owing to the slow, obscure, and insidious character of the disease, the mental affection has generally been of some duration before the attention of the practitioner has been directed to its existence. As this form of derangement generally exhibits itself in trifling perversions of the affections and propensities, leading to little acts of extravagance and irregularity of conduct, associated with great depression, we often find the attack has existed some years before a necessity has been felt for any medical advice or treatment—perhaps a suicidal propensity has manifested itself, this being the first apparent overt act of insanity.

It is necessary, before suggesting any course of treatment in melancholia, to ascertain whether any latent visceral disease be present. Occasionally, the local irritation will be found either in the liver, the stomach and bowels, or uterus. In the religious and other forms of melancholia in females, the delusions are often associated with uterine irritation; and under such circumstances, if actual physical derangement of an active character exists in this organ, the best treatment will be, the application of leeches to the neighbourhood of the uterus, combined with warm hip-baths, sedatives, and mineral tonics. In cases of melancholia, the digestive functions are often much deranged, the circulation, languid, the skin cold and flaccid, the secretion vitiated. These symptoms are often conjoined with a general loss of the *vis vitæ*. Such patients require generous diet, good air, gentle exercise, and occasional stimuli. When dyspeptic symptoms are combined with an inactive state of the bowels, I have often administered the compound tincture of guaiacum with great benefit. It is important to watch the particular features in these cases, and to improve the general health by the exhibition of mild alteratives and vegetable tonics, with alkalies. I have occasionally administered, with success, in this form of insanity, apparently associated with an abnormal condition of the nutrition of the brain, cod-liver oil, with preparations of iron.

My time will not admit of my submitting for your approval the treatment best adapted for those forms of the mental disease associated with an atrophied or softened condition of the nervous matter. I think more is to be done for the cure of these cases than the writings of medical men would lead the student to suppose, particularly if the disease be seen and subjected to treatment in the early stages. I have recorded the details of several instances of cerebral disease, exhibiting all the legitimate features of ramollissement, and yielding to the persevering administration of the preparations of iron, phosphorus, zinc, and strychnia, combined with generous living, and the occasional application of a leech behind the ear, should indications of cerebral congestion be present.* I have also derived benefit from the use of the milder forms of mercurials, associated with cinchona. In cases of impairment of the mind, loss of memory, defective power of attention, occasional paroxysms of *mental* paralysis, unconnected with lesions of the *motor* power, I have found a solution of the acetate of strychnine, and a solution of the phosphate of strychnine, of great advantage.

In some chronic forms of insanity, in dementia, and persistent monomania, connected, as it was supposed, with morbid thickening of the dura mater, and with interstitial infiltration of the membrane, as well as with exudations upon its surface, I have occasionally had the head shaved, and have perseveringly rubbed over the scalp a strong ointment of the iodide of potassium combined with strychnine. In other instances I have kept the head painted with the mixture of iodine. I have seen marked benefit from this mode of treatment. When the mental symptoms are supposed to be associated with effusions of serum, I have ordered the iodine to be applied externally, at the same time exhibiting minute doses of calomel, or mercury-with-chalk, to slightly affect the system: this, conjoined with occasional tonics, diuretics, and stimuli to support the vital powers, is occasionally productive of considerable benefit, in cases apparently placed quite beyond the reach of improvement or cure.

I have briefly referred to two distressing and often unmanageable forms of insanity—viz., of *suicidal mania*, and of those cases where the *patient obstinately refuses to take either food or medicine.* In insanity associated with suicidal tendencies, it is important to ascertain whether any cerebral congestion exists. If such be the case, a few leeches applied to the head, followed by an active cathartic, will relieve the local irritation, and often dissipate the idea of self-destruction. In the absence of any positive active cerebral symptoms, the prolonged hot bath, and the persevering exhibition of some form of sedative, is the best treatment to be adopted. I have seen the suicidal impulse removed after the administration of a few doses of belladonna; but the meconite and hydrochlorate of morphia, if given for a sufficient length of time, will, in the great majority of cases, distinct from actual incurable visceral or cerebral disease, effect a cure. Occa-

* In 1830, twenty-four years ago, my first observations on "Ramollissement of the Brain" were published in the *Lancet.*

sionally, the shower-bath, and counter-irritation in the vicinity of the head, will aid us in re-establishing health. Cases sometimes present themselves where the patient obstinately refuses to take either food or medicine. This character of case gives much anxiety. The refusal of food may be connected with the intention to destroy life, or it may be associated with and caused by delusive impressions. I am inclined to believe, that, in the majority of these cases, the symptom is the result of some irritation existing in the great ganglionic centres remote from the sensorium, affecting by direct action the organ of thought. Upon examination, we often find, in these cases, great gastric derangement, obstinate constipation, considerable tenderness upon pressure in the epigastric region, hepatic disease, the tongue foul, breath offensive, and other symptoms of derangement of the chylopoietic viscera. The determination to resist nourishment arises, under such circumstances, from a *positive loathing of food—a want of all inclination for it.* I have seen cases where it has been deemed necessary, in order to prolong life, to introduce food forcibly into the stomach, speedily cured by the adoption of means calculated to improve the general health and digestive organs. Mild alteratives, vegetable tonics, blisters over the region of the stomach, if the patient complain of pain in that region upon pressure, the warm and shower bath, are the most successful remedies to adopt in cases connected with obvious visceral derangement. Instances sometimes occur, where the refusal of food is clearly traceable to the presence of a delusion—an hallucination of taste, which makes everything appear to the patient bitter, disgusting, and poisonous. The unhappy patient often imagines that he is commanded, either by good or evil spirits, not to eat. These patients must be treated upon general principles, and the remedies be adapted to the peculiar character of each individual case. Under such hallucinations of taste, patients often swallow the most extraordinary articles. The case of a lunatic is recorded, who imagined that his stomach required to be strengthened with iron. He was seized with inflammation of the œsophagus, of which he nearly died. He then confessed that he had swallowed the blade of a knife. After his death, there was found in his stomach seven oxidated lath nails, each two inches and a half long; thirty-three nails, two inches long; forty-nine smaller iron nails and rivets; three pieces of wound-up iron wire; an iron screw, an inch long; a brass image of a saint; part of the blade of a knife; and other articles; amounting in number to 100, and weighing about twenty ounces. It will be necessary, in cases like those to which I have been referring, to ascertain whether the determination not to eat is the effect of such perversions or hallucinations of taste.

I can only in this lecture allude in general terms to the importance, as a principle of treatment, of the administration of tonic remedies, active exercise in the open air, and to good and generous living. It is rarely necessary, in the treatment of insanity, to deprive the patient of animal food. Individual cases occasionally come under our notice, in which it is indispensable, for a time, to enforce a farinaceous diet; but such is not often our duty. Among paupers, insanity is frequently cured by the free use of good animal food, and a generous supply of porter. Even when we are satisfied of the necessity of local depletion, it will often be requisite to give wine, and allow the patient a generous diet.

There are many other essential points in connexion with this important, this vast subject, which I am reluctantly compelled to pass entirely over. When I had resolved to bring this matter before the profession, I quite despaired, in the time allotted for *one* lecture, of being able to skim even upon the surface of the many deeply interesting points involved in the inquiry; but feeling—deeply, earnestly feeling—that, in relation to my own speciality, the subject of the medical treatment of insanity was of the first moment, of the most vital importance, to the profession as well as to the public, I did not hesitate in selecting this topic for one of my lectures, feeling assured that you would kindly make allowance for all imperfections, and generously appreciate the difficulties I had to encounter in concentrating in one short lecture a faint glimpse or shadow of a subject requiring for its successful exposition nine or ten lectures, equal in length to the one I have had the honour of reading this evening. I may have formed an extravagant and exaggerated conception of this subject, but I cannot close my eyes to the *fatal consequences which have so often ensued from a belief in the incurability of insanity by medical means.* In all grades of society we witness the pernicious, the fatal, the disastrous effects of this dogma. We see it influencing the conduct of county magistrates in the architectural proportions, medical organization and general arrangements of our great national asylums. We also perceive the consequences of the error operating in many of the private institutions for the treatment of the insane, thereby degrading them into places of detention, instead of conferring upon them the character of HOSPITALS FOR THE CURE OF THE INSANE, under the supervision of medical officers, well trained, by preliminary education, for their important vocation, acquainted with the philosophy of the human mind, and fitted by the character of their *heart,* as well as by the vigour of their *intellect,* for the right performance of the solemn and responsible duties entrusted to them by the public and the legislature.

